# Autophagy-Dependent Secretion: Contribution to Tumor Progression

**DOI:** 10.3389/fonc.2016.00251

**Published:** 2016-11-25

**Authors:** Tom G. Keulers, Marco B. E. Schaaf, Kasper M. A. Rouschop

**Affiliations:** ^1^Maastricht Radiation Oncology (MaastRO) Lab, GROW – School for Oncology and Developmental Biology, Maastricht University Medical Center, Maastricht, Netherlands; ^2^Cell Death Research and Therapy (CDRT) Laboratory, Department Cellular and Molecular Medicine, KU Leuven, University of Leuven, Leuven, Belgium

**Keywords:** autophagy, secretion, autosecretion, tumor progression, tumor microenvironment, unconventional secretion

## Abstract

Autophagy is best known as a lysosomal degradation and recycling pathway to maintain cellular homeostasis. During autophagy, cytoplasmic content is recognized and packed in autophagic vacuoles, or autophagosomes, and targeted for degradation. However, during the last years, it has become evident that the role of autophagy is not restricted to degradation alone but also mediates unconventional forms of secretion. Furthermore, cells with defects in autophagy apparently are able to reroute their cargo, like mitochondria, to the extracellular environment; effects that contribute to an array of pathologies. In this review, we discuss the current knowledge of the physiological roles of autophagy-dependent secretion, i.e., the effect on inflammation and insulin/hormone secretion. Finally, we focus on the effects of autophagy-dependent secretion on the tumor microenvironment (TME) and tumor progression. The autophagy-mediated secreted factors may stimulate cellular proliferation *via* auto- and paracrine signaling. The autophagy-mediated release of immune modulating proteins changes the immunosuppresive TME and may promote an invasive phenotype. These effects may be either direct or indirect through facilitating formation of the mobilized vesicle, aid in anterograde trafficking, or alterations in homeostasis and/or autonomous cell signaling.

## Autophagy

Autophagy is a catabolic process required to maintain cellular homeostasis by lysosomal degradation of aged/damaged organelles (e.g., mitochondria), protein aggregates, and pathogens. Autophagy commences with the formation of an initial cup-shaped membrane (phagophore) that elongates while (non-) selectively capturing cytoplasmic constituents into a double-membrane vesicle (autophagosome). Ultimately, the autophagosome fuses with a hydrolase and protease containing lysosome for degradation of the content. The end-products are recycled into the cytosol and are reused in processes including protein synthesis and ATP production.

During the last decade, extensive research revealed that at least 38 autophagy-related proteins (ATGs) comprise the core autophagy machinery that mediate initiation, elongation, cargo recruitment, and fusion with lysosomes (1). Furthermore, the yeast atg8 orthologs of the LC3/GABARAP protein family fulfill specialized roles in the execution of autophagy (2). This family consists of seven active members [LC3A (two splicing variants; LC3A-a and LC3A-b), LC3B, LC3C, GABARAP, GABARAPL1, and GABARAPL2], which share a high mutual homology, including a conserved C-terminal glycine residue for phosphatidylethanolamine (PE) conjugation to allow membrane association. Conjugation of the LC3/GABARAP protein family members to PE is required for expansion and closure of the phagophore. This process is controlled by two ubiquitin-like conjugation systems, a process closely orchestrated the ATG4, -7, and -3 and the ATG12–ATG5–ATG16L1 complex (3).

Despite the high grade of homology, the protein family members harbor individual roles in autophagy and are associated with autophagy unrelated functions, such as receptor trafficking, too (2).

Autophagy (and related defects) is associated with several pathologies, including neuropathologies, Crohns disease, and cancer. In cancer, autophagy is considered a double-edged sword, i.e., in healthy tissue, autophagy plays a cytoprotective role by maintaining homeostasis through degradation of cytotoxic constituents, which otherwise may trigger tumorigenic events. Nevertheless, once a tumor is formed, autophagy contributes to survival of cancer cells in areas deprived of nutrients of oxygen (hypoxia) (4, 5), a common feature of solid tumors that contributes to tumor progression, therapy resistance, and metastases formation (6).

Yet, accumulating research shows that the homeostatic role of autophagy and its related proteins is more elaborate than the degradation of cytoplasmic content alone. Autophagy not only contributes to intracellular homeostasis but also seems to contribute to tissue homeostasis by mediating intercellular communication. Peptides, proteins, and hormones that fail to enter the conventional secretory system due to the lack of a leader/secretion signal sequence can be secreted in an autophagy-dependent manner.

In this review, we list the current knowledge on the role of the autophagy machinery in autophagy-dependent secretion and specifically focus on factors that may influence tumor progression.

## (UN)Conventional Protein Secretion

In eukaryotes, a classical route for proteins to be secreted is the endoplasmic reticulum (ER)-to-Golgi route. Herein, proteins are directed toward the ER by their amino terminal signal peptide (leader peptide) and progress through vesicular flow to the Golgi. In brief, after ER translocation, proteins are oligomerized and packed into carrier vesicles that exit the ER at specialized regions. These vesicles assemble into vesiculo-tubular structure intermediates known as the ER-to-Golgi intermediate compartments (ERGIC) that, by lateral communication, sort proteins for further anterograde flow to the Golgi complex. In the Golgi, proteins are glycosylated to ensure proper protein structure, increase stability, and to allow interactions with target proteins (7). In the trans-Golgi network, secretory proteins are sorted into secretory vesicles that deliver their content to the plasma membrane to result in secretion (8). An increasing number of secreted proteins that lack the leader peptide have been identified. These proteins require alternative pathways to be secreted in a regulated fashion. This implies differences in vesicle formation, sorting, and transportation. Multiple alternate, non-classical pathways exist and are commonly referred to as unconventional protein secretion and include both non-vesicular and vesicular mechanisms (9). For example, cytosol-residing proteins, including ABC transporter-mediated yeast a-factor (3) or mammalian fibroblast growth factor 2 (FGF2) are directly transported across the plasma membrane. For FGF2 secretion, this is presumed to be mediated through PI(4,5)P_2_-induced oligomerization followed by membrane insertion and translocation (10). Other proteins are sorted into vesicular intermediates that fuse with the plasma membrane to release their content into the extracellular space [interleukin 1β (IL-1β) and IL-18 (11)], in part dependent on proteins required for autophagy execution (12, 13). This suggests that autophagy-proteins are required to produce secretory vesicles or that autophagosomes gain exocytic features. These processes in autophagy-mediated secretion are distinct from its degradative compartment [as reviewed in more detail in Ref. (14)]. In this context, the trafficking, tethering, docking, and plasma membrane fusion events would differ from canonical autophagy and resemble exocytic mechanisms. Herein, secretory vesicles are diverted from the endocytic system to be released from multivesiclular bodies (MVB). These secretory vesicles are then transported to the plasma membrane for content release. For example, the interaction between autophagosomes and the endocytic/MVB pathway [reviewed in Ref. (15)] is required for acetyl-CoA-binding protein (Acb1)-secretion and requires the fusion of Acb1-containing autophagosomes with endosomes or MVBs before plasma membrane fusion (16).

The involvement of autophagy-related proteins, in protein secretion indicates that cells utilize these effectors in a surprising mechanism of unconventional secretion. Here, we will discuss the data that support a relation between autophagy or autophagy-related proteins with secretory pathways (Table 1). First, we will elaborate on the physiological roles of autophagy in secretory events. Second, cancer cells utilize secretory mechanisms to communicate with stroma and surrounding cancer cells, thereby inducing alterations that influence tumor progression, including immunoevasion, immunesuppression, auto- and paracrine signaling, and angiogenesis.

**Table 1:** **Autophagy-dependent secreted factors**.

Protein	Mechanism	Reference
Acbp	Atg5, Atg7, Atg8, Atg12	(16)
IL-1B	ATG5	(11)
NBR1, P62, OPTN, and CACO2 LC3B-II	Inhibition or depletion of PIKfyve by apilimod or siRNA	(19)
Angiogenin 4, interlectin 1, and relmβ	ATG16L1 ATG5 or ATG7	(25)
MUC5AC	ATG5 and ATG16L1	(26)
Insulin	GABARAP	
NPY	ATG16L1 as RAB33a effector	(33)
von Willebrand factor (vWF), P-selectin, interleukin-8, angiopoietin-2, and endothelin-1	ATG7, ATG5, chloroquine or bafilomycin A1	(34)
Matrix-degrading factors including cathepsin K	ATG5 or ATG7	(35)

## Physiological Roles of Autophagy-Mediated Secretion

The first evidence for the involvement of autophagy-related proteins in protein secretion was obtained in yeast. Acyl coenzyme A-binding protein (Acbp) is secreted through an unconventional secretory pathway that depends on components of the autophagic process (Atg5, Atg7, Atg8, Atg12) (16). Importantly, this study also demonstrated that autophagy connects with compartments (multivesicular bodies) of endosomal trafficking and SNARE-dependent membrane fusion events are both required for the Acbp secretion. Since the discovery of autophagy-dependent secretion in yeast, also several mammalian factors have been identified to be dependent on autophagy-related proteins for their secretion.

Phosphoinositides are membrane lipids with specific recognition domains for the recruitment of cytosolic proteins and are involved in the regulation of membrane transport. So far, seven phosphoinositides have been described. They can be converted into each other by phosphoinositide kinases and phosphatases (17). PIKfyve is one of those converting kinases and has been implicated in membrane transport events. The substrate of PIKfyve, PI(3)P, plays a critical role in the initiation of autophagy and autophagosome maturation (18). Inhibition or depletion of PIKfyve by apilimod or siRNA resulted in decreased autophagic flux, probably caused by an impaired autophagosome/lysosome fusion. Interestingly, prostate cancer cells incubated with the PIKfyve inhibitor apilimod, secrete more autophagy-associated proteins (NBR1, P62, OPTN and CACO2, and membrane-bound LC3B); however, proteins involved in earlier stages of autophagy were not observed (ATG2A, ATG5, and ATG16L). Analysis of density gradients reveal that these autophagy-associated proteins are expressed on vesicles and appear as autophagosome subpopulations, suggesting induced secretory autophagy (19).

### Cytokines and Inflammation

Autophagy is able to suppress inflammasome activation through maintaining mitochondrial homeostasis (20). In line, Zhang et al. observed increased inflammasome activation in macrophages after sepsis in Gamma (γ)-aminobutyric acid type A receptor (GABAAR)-associated protein (GABARAP) knockout mice (20). Secretion of cytokines is central in inflammasome activity. Well-documented cytokines that are associated with unconventional secretion in an autophagy-dependent manner are interleukin-1 beta (IL-1B) and IL-18. Dupont et al. demonstrated in bone marrow-derived macrophages during conditions of induced autophagy (starvation or Torkinib treatment) that secretion of IL-1B increased, which was reduced in the absence of ATG5. Consistent with data obtained from yeast Acbp, similar additional factors were required [as mammalian equivalents of yeast Golgi-associated protein (GRASP55) and small GTPase Rab8a], indicating a conserved system or cooperation (11).

Interestingly, three-dimensional STochastic Optical Reconstruction Microscopy (STORM) demonstrated that IL-1B was not only present in the inner vesicle of the autophagosome but was already recruited to the emerging phagophore. In this, IL-1B is actively translocated across the membrane and accumulated in the intermembrane space of the mature autophagosome (21). This, in contrast to the sequestration of degradable autophagosomal content which is located in the inner vesicle as a result of cytoplasmic engulfment and closure of the phagophore, novel mechanism indicates differences in cargo recruitment and indicates formation of different vesicles by the autophagy-related proteins that may guide the distinct fate of the produced vesicle.

### Role of Autophagy-Dependent Secretion in Establishing a Barrier for Infection

Paneth cells are specialized epithelial cells present at the base of the crypts of Lieberkuhn. These cells control the intestinal microbiota through secretion of intracellular granules that contain antimicrobial peptides and lysozyme. Autophagy-proteins are essential for Paneth cell function. Knockdown of crucial autophagy genes [ATG16L1, ATG5, or ATG7 (22, 23)] result in alterations in Paneth cell granules, as illustrated by fewer and aberrant granules with diffuse lysozyme patterns and decreased presence of lysozyme in mucus. Due to its relevance in establishing a microbial barrier, the ATG16L1 gene was annotated as a Crohns disease (CD) risk allele (24) and the phenotype is similar to the abnormalities found in Paneth cells of CD patients.

In contrast to CD where the ileum and colon are often affected, ulcerative colitis is restricted to the colon only. Conditional knockdown of ATG7 in the colon (GlcNAc6ST-2-Cre) increased bacterial colonization in crypts. This phenotype was associated with a decreased release of mucin from goblet cells and reduced expression of antimicrobial and antiparasitic peptides (angiogenin 4, interlectin 1, and relmβ). Hence, colonic ATG7 knockdown results in increased susceptibility to the development of UC-like colitis (25).

In line with autophagy-dependent secretion by intestinal tissue, ATG5 and ATG16L1 deficiency results in reduced secretion of mucins (MUC5AC) by human tracheal epithelial cells (26).

Together, autophagy-dependent secretion is required to maintain effective antibacterial barriers by epithelial cells. In line, ATG16L1 conditional knockout mice (Villin-Cre) are more susceptible to *Salmonella typhimurium* infection (27). Although, beyond the contribution of establishing a barrier function, autophagy is required for limiting bacterial proliferation through ATG16L1- and LC3C-dependent degradation of cytosol-residing *Salmonella* (xenophagy) (28). Evidently, autophagy is a prerequisite for different cell types to maintain their function (e.g., mucus secretion) thereby and protection against inflammatory disorders.

### Insulin Secretion

Pancreatic β-cells are specialized in secretion of insulin in response to high levels of blood glucose concentrations. Release of insulin is mediated by the fusion of insulin-containing vesicles with the plasma membrane. These insulin-containing vesicles are transported toward the plasma membrane. GABARAP, one of seven mammalian variants of yeast Atg8, which is already known to be implicated in multiple cellular functions including fusion events in autophagy (29) and receptor trafficking (30), has recently been demonstrated to mediate insulin secretion. Herein, GABARAP binds insulin-containing vesicles in a PE conjugation-dependent mechanism (31). Together with its microtubules-binding feature [residues 10–22 (30)], GABARAP allows vesicle presentation to the motor protein KIF5B (located at microtubules) and facilitates vesicle mobility and insulin release (32).

### NPY Secretion

In rat adrenal medullar pheochromocytoma derived cells, the neurites display ATG16L at dense-core vesicles. Although these vesicles do not co-localize with LC3, release of peptide hormone neuropeptide Y (NPY) is dependent on ATG16L1 functioning as a Rab33a (which regulates membrane trafficking events) effector. Interestingly, NPY secretion was not altered in cells that were deficient in autophagy by overexpressing ATG4B^C74A^ or knockdown of ATG13 or ULK1 and indicates a highly specialized role for ATG16L1 in hormone secretion that is independent of other canonical autophagy-associated proteins (33).

### Autophagy-Mediated Secretion of Weibel–Palade Bodies in Vascular Endothelial Cells

Vascular injury stimulates endothelial cells to secrete factors to promote repair. Endothelial secretory granules [Weibel–Palade bodies (WPBs)] contain active molecules, including von Willebrand factor (vWF), P-selectin, interleukin-8, angiopoietin-2, and endothelin-1. The intracellular WPBs are characterized by striations parallel to its longitudinal axis and are delineated by a membrane. When secreted, the multimeric hemostatic vWF is tethered to the connective tissue to mediate platelet adhesion at sites of vascular injury. The processing of vWF to a mature form is important in the WPB formation. Treatment with chloroquine or bafilomycin A1, both raise lysosomal pH, and knockdown of ATG7 or ATG5 reduce the number of endothelial WPBs due to incorrect processing of vWF. In line, endothelial specific deletion of *Atg7* in mice led to reduced epinephrine-induced plasma vWF levels and increased bleeding time. Thus, autophagy in endothelial cells aids in hemostasis by proper maturation of WPBs (34).

### Osteoclastic Bone Resorption

Bone remodeling is a lifelong process of bone degradation and formation important for bone healing after injury, bone restructuring, and sustaining bone homeostasis. Osteoclasts cooperate with osteoblasts in bone remodeling in which osteoclasts are responsible for bone degradation and reabsorption of mineralized bone matrix. The osteoclasts are large multinucleated cells that are characterized by their ruffled border by which contact area with the bone is increased. Fusion of secretory lysosomes with the ruffled border causes release of matrix-degrading factors including cathepsin K which aids bone matrix degradation. ATG5- or ATG7-deficient osteoclasts lack a normal ruffled border, impaired localization of secretory factors, including cathepsin K, and eventually have impaired bone resorption. Importantly, development of osteoclasts was not aberrant indicating that the autophagy deficiency led to functional impairment independent of sustaining cellular homeostasis. Moreover, secretory lysosome formation was unaffected, whereas Rab7 (key factor for lysosome fusion events) localization to ruffled border was ATG5 dependent. Together, these data indicated that autophagy-related proteins aid in secretory events at the osteoclast ruffled border by directing fusion of the secretory lysosome with the plasma membrane (35).

In conclusion, autophagy is important for secretory functions of various cell types. Important to note is that autophagy can have either direct or indirect contribution to protein secretion. For example, ATGs directly facilitate protein secretion by mediating cargo sequestration (as IL-1B) or vesicle trafficking (as insulin), but autophagy also maintains cellular homeostasis that is important to preserve the specialized function (IL-1B and vWF) and primes the plasma membrane for proper release of autophagy-independent secretory vesicle (as in osteoclasts). Furthermore, for sustaining a microbial barrier, autophagy’s contribution is dual as both a canonical form of autophagy (xenophagy) and the regulation of important secretory factors as mucin contribute to prevent pathogen invasion.

## Secretory Autophagy: Waste Disposal?

Damaged and aggregated proteins and aged organelles are typically degraded by autophagy. Substrates for autophagy are ubiquitylated and recognized by autophagy receptors and degraded. Recent work indicates that defective or saturated autophagy, i.e., by defective autophagosome/lysosome fusion results in cargo secretion into the extracellular environment.

Lysosomal dysfunction is associated with the secretion of aggregation prone proteins that are associated with neurodegenerative diseases as Parkinson’s and Alzheimer’s disease. α-Synuclein is a presynaptic neuronal protein that is genetically and neuropathologically linked to Parkinson’s disease. Wild-type α-synuclein is typically degraded by the autophagy and the proteasome (36). Interestingly, tubulin polymerization-promoting protein/p25a, expressed in the CNS, sorts α-synuclein into autophagsomes but simultaneously prevents its degradation through inhibition of autophagosome/lysosome fusion. Instead p25a controls α-synuclein clearance by its release in the extracellular environment in an autophagy-dependent manner (37).

Alzheimers’ disease is characterized by the accumulation of intracellular Amyloid beta (AB) peptide and tau aggregates and extracellular AB plaques. In normal conditions, intracellular proteins are cleared by autophagy and autophagosomes are resolved in the process. However, during Alzheimer disease, autophagosomes accumulate, indicative of impaired autophagy. Autophagy deficiency (ATG7 knockout) in excitatory neurons results in intracellular AB accumulation, confirming its role in clearance of AB aggregates by autophagy. Although AB increased intracellularly, extracellular AB plaque formation was drastically reduced. Reconstitution of ATG7 expression by lentiviral transduction, rescued the secretory phenotype. In parallel, pharmacologic modulation by either induction or inhibition of autophagy, by rapamycin or spautin-1, increased and reduced extracellular AB release, respectively. Thus, autophagy influences intracellular transport and secretion of AB (38).

Mitochondria, the energy producing centers of the cell, generate ROS as a byproduct of oxidative phosphorylation. In many cancers, ROS production is increased due to mutations in mitochondrial DNA, hypoxia, or disturbed metabolism, leading to cancer progression (39). The homeostasis of mitochondrial ROS plays an important role in the regulation of autophagy. Depolarized and ROS leaking mitochondria are typically degraded by a selective form of autophagy, mitophagy. In depolarized mitochondria, PINK recruits Parkin to mediate selective removal of the organelle in a degrative autophagy-dependent manner. However, a recent report shows that there is an alternative way to maintain mitochondrial homeostasis in the cell. Mesenchymal stem cells (MSC) pack depolarized mitochondria in microvesicles and release them in the extracellular environment to outsource mitophagy where they are recognized by and transferred to macrophages. These released microvesicles are highly enriched in LC3 and ATG12 compared to whole-cell extracts. This mitochondrial transfer probably serves to increase MSC survival (40). Similarly, lipopolysaccharide (LPS)-stimulated rat hepatocytes secrete mitochondrial proteins CPS1 and COXIV, a component of the mitochondrial respiratory chain and associated with the inner mitochondrial membrane, and mitophagy-related proteins PARK2, and PINK1 and LC3B-II. These effects are inhibited by the autophagy inhibitor 3methyladenine or after *Atg5* knockout, suggesting a role for autophagy in the secretion of mitochondria after LPS stimulation (41).

Also endothelial cells are able to release vesicles with autophagosome characteristics. During apoptosis, endothelial cells release in addition to apoptotic bodies, vesicles in an unconventional manner (13). Ultrastructural analysis by electron microscopy showed single membrane vesicles up to 10 μm which contained structures of mitochondria, multivesicular bodies, and autophagosomes. Further proteomic analysis revealed the release of autophagy-associated proteins ATG16L1, LAMP2, and LC3B. The biological function is of this phenomenon remains to be elucidated (12).

The previous section lists the evidence that cells are able to release autophagic vesicles into the extracellular environment. Vesicle release during defects in the autophagic process, specifically during autophagosome/lysosome fusion, suggests alternative mechanisms in waste removal.

## Effects on the Tumor Microenvironment

Regardless of the clinical advances in the past decades that have improved cancer patient outcome, cancer is still one of the leading causes of death in the world. Importantly, the efficacy of treatment strategies is heavily influenced by cancer cell autonomous features but also by the tumor microenvironment (TME). Solid tumors consist of a variety of cell types, including the cancer cells, endothelial cells, immune cells, and fibroblasts and contain well- and poorly perfused areas that results in inefficient nutrient and oxygen supply (42, 43). Normal (non-transformed) cells in the TME are reprogrammed by the cancer cells to their benefit. This is exemplified by growth supporting angiogenesis and the suppression of anti tumor immunity. Importantly, an existing connection between autophagy and tumorigenesis has already been established. For example, deletion of a single *BECN1* allele [Beclin1 protein important regulator of autophagy (44)] predisposes mice to spontaneous tumor development (45, 46). Further, depletion of FIP200 (important for autophagy initiation) in mammary cancer cells inhibits tumor initiation and progression including metastases (47). Autophagy in cancer cells supports their survival (by aiding the high energy demand) and abets resistance to metabolic and oxidative stresses (e.g., hypoxia) (4, 48–52). Although this role is well established, the contribution of autophagy-related intercellular communication that influences tumor progression through evasion of immunosurveillance, immunogenic cell death (ICD), angiogenesis, and an invasive phenotype is an emerging field with great interest. For example, in cancer, the RAS genes HRAS and KRAS are frequently mutated. Although the exact role of autophagy in tumor progression of RAS-mutated tumors is still under debate, autophagy seems to be dispensable for the growth and survival of KRAS-mutated cancer cell lines derived from human tumors (53). However, when non-RAS-mutated cells are transformed with oncogenic RAS, these cells are highly dependent on autophagy for tumorigenic events (54). In addition, the invasive phenotype of HRASV12-transformed breast cancer cells is reduced in ATG7 knockdown cells. This invasive phenotype could be rescued by incubating these autophagy-deficient cells with conditioned medium of autophagy-proficient cells. This supports an autophagy-dependent secretory system that supports tumor progression (55).

In the next section, the current knowledge on proteins secreted through autophagy-mediated processes that influence tumor progression is discussed (Table 2).

**Table 2:** **Effects on the tumor microenvironment**.

Protein(s)	Mechanism	Effect	Reference
**Influencing immunogenic cell death to evade immune surveillance**			
ATP	ATG5 knockdown	After radiotherapy and MTX exposure “eat me signal” for immune cells. Stimulus for DC recruitment, IFNγ-producing CD4 and CD8 T cells	(60–62)
HMGB1	ATG5^fl/fl^	Promote processing and presentation of tumor antigens by DCs, enhanced immuno surveillance	(11)
**Cytokine release and influence on the tumor microenvironment**			
IL1-B, IL-6, IL2	GABARAP Knockout mice	Increased secretion by macrophages	(78)
IFNγ	GABARAP Knockout mice	Increased secretion by lymphocytes	(78)
CXCL9, CXCL10, and CXCL11	FIP200 conditional knockout	Enhanced secretion, leading to improved immuno surveillance	(47)
**Prometastatic: driving an invasive behavior of cancer cells**			
LIF, FAMC3, DKK3, IL-8	ATG7 knockdown	These factor promote metastasis *via* MMP2 upregulation (IL-8), epithelial to mesenchymal transition (FAM3C, DKK3, LIF), and promotion of angiogenesis (IL-8 and DKK3)	(80)
	ATG7, ATG12, ATG3 knockdown chloroquine or bafilomycin A1	Autophagy-deficient HRAS^V12^-transformed breast cancer cell lines display reduced invasive protrusions. Conditioned medium of autophagy-proficient cells rescued the invasive phenotype	(79)
IL6, CCL-2, CCL-20, VEGFA, MMP2	3-MA, ATG5, and ATG7 knockdown	TLR3 and TLR4 activation leads to autophagy-dependent secretion of these factors associated with a migratory and invasive phenotype of lung cancer cells	(82)
IL6	ATG7 or beclin knockdown	Autophagy deficiency lead to an increase or decrease in low or high autophagic breast cancer cells, respectively. Autophagy-dependent secretion of IL-6 are able to promote mammosphere formation and may be important in CSC maintenance	(83, 84)
**Prometastatic: proper Weibel–Palade body formation in vascular endothelium to facilitate metastasis**			
WPB proteins		Autophagy is important to sustain secretion of WPBs containing proteins that influence tumor progression	(85)
**Chemoresistantance**			
HMGB1		HMGB1 causes doxorubicin resistance in neighboring breast cancer cells	(88)

### Influencing Immunogenic Cell Death to Evade Immunosurveillance

Under normal circumstances, immune cells [including dendritic cells (DCs), natural killer (NK) cells, and T cells] recognize and eliminate newly formed neoplastic cells due to their high immunogenic nature as a result of their mutational burden (immunosurveillance). Cancer cells that have obtained an immune evasive phenotype are able to circumvent recognition and subsequent elimination by the cooperative immune cells. These cancer cells are then selected for characteristics that circumvent local immunosurveillance and contribute to the growth of the lesion. With recent advances, the immune evasive feature is a topic of interest for the development of therapeutic strategies. Ideally, the elicited cancer therapy-induced cell death should provoke an immunogenic chain reaction that includes boosting the immune system to tilt the balance toward recognition rather than evasion, called ICD. ICD invokes the release of immunomodulatory proteins [damage-associated molecular patterns (DAMPs)] that incite antitumor immunity (56). ICD can be induced by selective chemotherapeutics, including mitoxantrone (MTX) and oxaliplatin (OXA) and radiotherapy [reviewed in Ref. (57)]. Cancer cells undergoing ICD stimulate and activate the innate immune cells. Subsequently, this can result in the cross-priming of the adaptive immune system for the antigens of dying cancer cells, thereby leading to an effective activation of antitumor immunity. This can elicit a long-term therapeutic effect (even after therapy has stopped) and is fundamental to observed abscopal effects. In line, clinical studies have demonstrated that lymphopenia negatively affects chemotherapeutic response of solid tumors (58) and that ICD-associated DAMPs can be used as predictive and prognostic biomarkers (59).

Important immunogenic DAMPs that are displayed by cells undergoing ICD are secretion of ATP, surface exposure of calreticulin (CRT), release of heat shock proteins, and high mobility group box 1 (HMGB1). Interestingly, ATG5 knockdown in colon cancer cells reduces ATP release after radiotherapy (60) and MTX exposure (61), which was associated with a decreased effect on tumor growth inhibition. Interestingly, in autophagy-deficient cells, no differences in CRT surface exposure or HMGB1 release were observed (61). The relevance of autophagy-dependent radiotherapy-induced ATP release was further supported by the observation that treatment with an inhibitor of ecto-ATPase only increased radiosensitivity in immunocompetent but not immunodeficient mice. Here, a partial rescue of lymphocyte infiltration indicates that the autophagy-dependent radiotherapy-induced ATP release enhances antitumor immunity (60). Similarly, for MTX treatment of osteosarcoma cells, it was also shown that ATG5 is required for ATP secretion. Interestingly, on a more mechanistic level, Martins et al. demonstrated that ATP (stored in lysosomes) is released upon MTX and OXA treatment and is associated with LAMP1 (lysosomal marker) translocation to the plasma membrane. Nonetheless, a role for autophagy seems to be maintaining an intracellular ATP pool (may even be cargo sequestration) required for (LAMP1+) lysosome-dependent ATP release. Accordingly, the replenishment of ATP to lysosomes was reduced when autophagy genes were knocked down (62). ATP release conveys an important “eat me” signal for immune cells. Once it is released, ATP may attract innate effector cells of the immune system into the tumor bed. Consistently, it was demonstrated that autophagy-dependent ATP release from MTX-treated colon cancers was a stimulus for DC recruitment, IFNγ-producing CD4 and CD8 T cells that had favorable effects on MTX sensitivity (61). Correspondingly, ATG7 in a genetically induced melanoma mouse model was required for MTX-dependent growth inhibition that was reliant on functional CD4 and CD8 T cells (63). Further, caloric restriction or treatment with caloric restriction mimetics, that increase autophagy activity, enhance autophagy-dependent ATP release and improve MTX-induced tumor growth delay in a T cell dependent fashion (64).

Oppositely, a different study using a different ICD inducer (photo-oxidative ER stress inducer hypericin) has demonstrated autophagy-independent ATP release, but observed enhanced surface CRT exposure when autophagy was attenuated. The enhanced DC maturation and IL-6 secretion further promote IFNγ-producing T lymphocytes (65). Interestingly, surface CRT exposure after MTX or hypericin treatment could be ablated in cells lacking lysosome-associated LAMP2A, an essential gene for a chaperone-mediated autophagy (66). Moreover, regarding ATP, it was demonstrated that extracellular residing ATP does not relay an immunogenic response *per se* (67) and further illustrates the context dependence of effects resulting in immunogenicity.

The involvement of autophagy-related proteins in the release of the DAMP HMGB1 has been demonstrated using ATG5^fl/fl^ Cre^+^ bone marrow-derived macrophages (11) and dying glioma cancer cells [in which HMGB1 was found in a subset of autophagosomes before release (68)]. The released HMGB1 by dying cancer cells can bind Toll-like receptor (TLR-) 4 and promote the processing and presentation of tumor antigens by DCs. This leads to cross-priming of T-cells and enhances immunosurveillance (69). Furthermore, endothelial cell exposure to HMGB1 triggers pro-angiogenic effects (70), including endothelial cell migration, sprouting and induction of an autocrine signaling cascade that results in elevated expression of leukocyte adhesion molecules ICAM-1, VCAM-1, and E-selectin. Moreover, HMGB1 induced expression of VEGF-A, VEGFR1, VEGFR2, and neuropilin-1 (71) and stimulation of angiogenesis (72).

In conclusion, ICD is an important pillar of therapy-induced antitumor immunity as it relays important signaling to the immune system, including DCs. DC stimulation may be important to induce tumor cure as demonstrated by ICD-based DC-vaccines in high grade glioma-bearing mice (73). Nevertheless, the influence of autophagy on the display of DAMPs may be ICD-inducer dependent and requires further understanding for effective use.

### Cytokine Release and Influence on the Tumor Microenvironment

ATP can bind the P2RX7 receptor and activate the NOD-like receptor family, pyrin domain containing 3 (NLRP3) inflammasome in DCs and macrophages (74). This inflammasome activation can be suppressed by autophagy (20), which would be favorable as inflammasome activation and subsequent IL-1B release by, e.g., macrophages have pro-tumorigenic effects. However, the P2RX7 receptor impairs autophagy by blocking lysosomal function and stimulates release of vesicles with autophagolysosome characteristics (75). This suggests that activation of the P2RX7 receptor leads to a secretory phenotype, *via* inhibition of autophagy. Depletion of IL-1B arrests growth in melanoma (76), and macrophage-derived IL-1B-induced IL-17 expression from γδ T cells resulted in expansion of tumor-associated neutrophils that suppress cytotoxic T cells in breast cancer, resulting in increased number of pulmonary and lymph node metastases (77). Alternatively, in a murine colon carcinoma model, autophagy-dependent ATP release after MTX treatment promoted recruitment of IFNγ-producing CD8+ T cells into the tumor in an IL-1B-mediated fashion (61).

Lipopolysaccharide/LPS + ATP stimulated GABARAP knockout macrophages to secrete more IL-1B and IL-6. In addition, GABARAP knockout lymphocytes produced more IL-2 and interferon-γ. In this model, GABARAP KO was associated with reduced tumor incidence. These effects were validated on tumor control in a melanoma tumor cell-inoculated model (78) and indicated that GABARAP in non-cancerous cells is sufficient to sustain pro-tumorigenic effects potentially due to control of cytokine secretion.

A study using MMTV-PyMT mouse model of breast cancer bearing a conditional deletion of autophagy gene *FIP200* shows that these tumor cells have a different chemokine secretion profile than FIP200-proficient cells. The TME polarizes toward an improved immunosurveillance as enhanced secretion of chemokines, including CXCL9, CXCL10, and CXCL11, leads to increased infiltration of IFNγ-producing CD8+ and CD4+ T cells (47).

In conclusion, autophagy-related secretion is important in controlling the cytokine profile of different cell types.

### Prometastatic: Driving an Invasive Behavior of Cancer Cells

Oncogenic mutations in RAS are highly prevalent in cancers and drive different pro-tumorigenic features, including proliferation, survival, and invasion. Autophagy-deficient HRAS^V12^-transformed breast cancer cell lines display reduced invasive protrusions in genetic knockdown models (including ATG7, ATG12, and ATG3) and after pharmacological inhibition (chloroquine or bafilomycin A1). Addition of conditioned medium of autophagy-proficient cells rescues the invasive phenotype, indicating a role for autophagy-dependent secretion in triggering cellular migration. Correspondingly, the pulmonary metastatic potential of HRAS^V12^ tumors is reduced in autophagy-deficient cells, effects dependent on autophagy-related secretion (55). Despite these results, in this model, the role of IL-6 in tumor progression is ambiguous in literature as both pro-tumorigenic (metastasis, angiogenesis, immune suppression) and antitumorigenic (CD8+ T cell trafficking to lymph nodes and tumors) effects on the TME are described (79).

Kraya et al. observed a cytokine profile that differed between melanoma cells with high and low autophagy activation that could be mimicked by introducing ATG7 knockdown in an autophagy high cell line. The main secretory factors the authors focused on, which were dependent on autophagy(-protein), are leukemia inhibitory factor (LIF), family with sequence similarity 3 member C (FAM3C), dickkopf WNT signaling pathway inhibitor 3 (DKK3), and IL-8. These factors are able to promote metastasis *via* mechanisms that include MMP2 upregulation (IL-8), epithelial to mesenchymal transition (FAM3C, DKK3, LIF), and promotion of angiogenesis (IL-8 and DKK3) (80).

Toll-like receptor 3 and TLR4, which are expressed on immune cells, including macrophages and DCs, but also a variety of cancer cell, can activate the release of an array of cytokines (81). Recently, it has been demonstrated that TLR3 and TLR4 activation [in an LPS- or poly(I:C)-induced model] in lung cancer cell lines (A549 and H460) causes (1) Lys63-linked ubiquitynilation of TNF receptor-associated factor 6 (TRAF6) and (2) induced autophagy. Herein, autophagy was required for TRAF6 ubiquitinylation that leads to downstream activation of NFκB and MAPK signaling and subsequent cytokine production. As a result, autophagy deficiency in these lung cancer cells reduced release of IL-6, C–C motif chemokine ligand (CCL)-2, and CCL-20. CCL-2 secretion is associated with cell migration and CCL-20 with a metastatic phenotype. Accordingly, autophagy deficiency impaired migratory capacity. IL-6 can induce VEGFA and MMP release that are associated with an invasive phenotype. Indeed, the invasive phenotype was dependent on autophagy and IL-6 and associated with VEGFA and MMP2 release (82).

In breast cancer, autophagy inhibition through ATG7 or Beclin1 knockdown altered IL-6 secretion. Interestingly, autophagy deficiency increased IL-6 secretion by MCF7 (low autophagy-dependent survival) and decreased IL-6 secretion by MDA-MB-468 (high autophagy-dependent survival) cells. IL-6 secretion is important for cancer stem cell (CSC) maintenance and is sufficient to induce CD44+:CD24low/− phenotype in breast cancer cells (83). In line, autophagy deficiency decreased mammosphere formation capacity of MDA-MB-468 cells. Rescue experiments illustrated that mammosphere formation was improved by IL-6 treatment and conditioned media from autophagy-proficient MDA-MB-468 cells. Autophagy-dependent secretion of IL-6, but also other factors, are able to promote mammosphere formation and may be important in CSC maintenance (84).

### Prometastatic: Weibel–Palade Body Formation in Vascular Endothelium to Facilitate Metastasis

Aberrant signaling in tumor-associated endothelial cells contributes to excessive neovascularization that is a feature of solid tumors [reviewed in Ref. (72)]. As discussed above, autophagy is important to sustain the secretion of protein-containing WPB by proper vWF maturation. In tumor endothelial cells (TECs), these WPBs contain important secretory factors that can influence tumor progression. In line, P-selectin is sorted into WPBs as a result of its ability to interact with vWF and is translocated to the cell membrane upon stimulation (85). Once localized at the luminal side of the endothelial cell, it facilitates metastasis formation by promoting adhesion of circulating tumor cells (86). Impairment of autophagy may, therefore, reduce development of metastases. In addition to vWF and P-selectin, WPBs can contain other secretory proteins as angiopoietin 2 which is positively associated with tumor progression (due to its angiogenic potential) and interleukin 8 which is important in tumor progression and metastasis (due to its angiogenic and immune response modulating potential). Collectively, autophagy-dependent WPB formation may facilitate tumor progression, although these aspects require further investigation.

### Therapy Resistance

Autophagy has been implicated in promoting chemo- and radioresistance. Although often presumed to be caused by its degradative feature, we demonstrated in irradiated cancer cells that knockdown of ATG7 or LC3B, but not treatment with lysosomal inhibitor chloroquine, sensitizes cancer cells to radiation (87). This further supports a role of autophagy-related proteins to promote radioresistance through an alternative process such as secretion. For example, HMGB1 is secreted through autophagy-dependent mechanisms during ICD (11, 68). Although this factor is an important DAMP that can increase immunogenic responses, HMGB1 increases doxorubicin resistance in neighboring breast cancer cells (88). In line, the interaction of HMGB1 with the receptor for advanced glycation end products (RAGE) that is expressed on various cell lines in the tumor increases chemo resistance by inducing pro-survival autophagy (89).

In addition to increasing angiogenesis and the prometastatic potential by DKK3, DKK3 expression is associated with docetaxel chemo sensitivity in lung cancer cells through decreasing expression of the drug efflux pump P-glycoprotein (90). Furthermore, DKK3 overexpression in an esophageal adenocarcinoma cell line was associated with increased 5-FU and cisplatin resistance, invasion, and activation of the TGF-B signaling (91).

In short, autophagy-dependent secretion is involved in antitumor effects through enhancing immunosurveillance, but is also important in tumor progression through stimulation of angiogenesis, changing drug resistance, triggering EMT, and increasing metastases development. Manipulation of the secreted arsenal of proteins, and tilting the balance more toward an antitumor strategy may be an attractive novel approach in cancer treatment.

## Autophagy-Dependent Receptor Trafficking in Tumor Progression

Autophagy execution requires cargo recognition, packaging, vesicle transport, vesicle fusion, and degradation. In addition to the catabolic function of autophagy, the autophagy machinery is utilized for more purposes, including intracellular trafficking and endocytic signaling. In addition to these roles in secretion, autophagy mediates the retro- and anterograde trafficking of membrane-bound receptors that may influence tumor progression.

For example, the GABARAP protein family mediates membrane of cell- surface expression of receptors like the GABA (A) receptor (GABAAR) (92), the human kappa opioid receptor (hKOPR) (93), transient receptor potential cation channel subfamily V member 1 (TRPV1) (94), the angiotensin II receptors (AGTR) (95, 96), and the epidermal growth factor receptor (EGFR) (51).

Epidermal growth factor receptor controls cell proliferation, migration and differentiation and is frequently overactivated in several cancer types due to amplification or mutation (97). EGFR expressing tumors depend on autophagy for their survival and proliferation. Inhibition of autophagy by the administration of chloroquine abrogated the radioresistant phenotype of these tumors (52) [reviewed in Ref. (98)]. Interestingly, during hypoxia, translocation of EGFR to the plasma membrane is controlled by GABARAPL1 (51). Upon hypoxia exposure, GABARAPL1 colocalizes with EGFR at the cytoplasmic site of the plasma membrane. Moreover, knockdown of GABARAPL1 resulted in a decrease in EGFR membrane expression, but not in overall EGFR expression, suggesting a role for GABARAPL1 in anterograde transport of EGFR.

The KOR and GABAAR are involved in neurological processes and play a role in a variety of processes like pain sensation, consciousness, and mood.

The GABA(A) receptor (GABAAR) is well known for its inhibitory role on active neurons and is expressed on the postsynaptic throughout the whole body, although mainly expressed in the mammalian brain. Surprisingly, overexpression of the GABAAR leads to several types of cancer including breast, liver, lung, and pancreatic cancers and contributes to migration of breast cancer cells through activation of extracellular-regulated kinase 1/2 (ERK1/2) (99–104). GABARAP and GABARAPL1 are both involved in GABAAR trafficking toward the plasma membrane. In this role, GABARAP probably serves as a cargo-receptor which mediates GABAAR incorporation in the transport vesicle by a direct interaction with the γ2 subunit of the receptor. GABARAP-knockdown mice show no defects in GABAAR expression, suggesting that GABARAP is redundant and other molecules, like its homolog GABARAPL1, can take over its function (105).

In contrast to the pro-tumorigenic effects of EGFR and GABAAR signaling, membrane expression mediated through autophagy-related proteins also results in the expression of receptors that may inhibit tumor progression, for example through KOR signaling. The KOR is well characterized for its analgesic role. However, the KOR also acts as a negative regulator of cell proliferation in breast, lung, and prostate cancers (106, 107). Opioid receptors belong to the GPCR family, and activation of the receptors modulates the MAPK pathway and inhibits prosurvival PI3K/AKT signaling molecules and may antagonize EGFR signaling (106). Both GABARAP and GABARAPL1 are required for anterograde transport of the KOR receptor. Interestingly, because of its stronger interaction, GABARAPL1 does not need C-terminal modification in contrast to GABARAP, which requires membrane association to transport the KOR to the plasma membrane (93).

Taken together, the GABARAP family proteins mediates trafficking and surface expression of receptors with both tumor promoting (EGFR, GABAAR) and tumor inhibitory characteristics (KOR). This suggests that the GABARAP family contributes to cancer progression in a context-dependent manner, being in a tumor-promoting or -inhibitory role.

## Concluding Remarks

**Figure 1:**
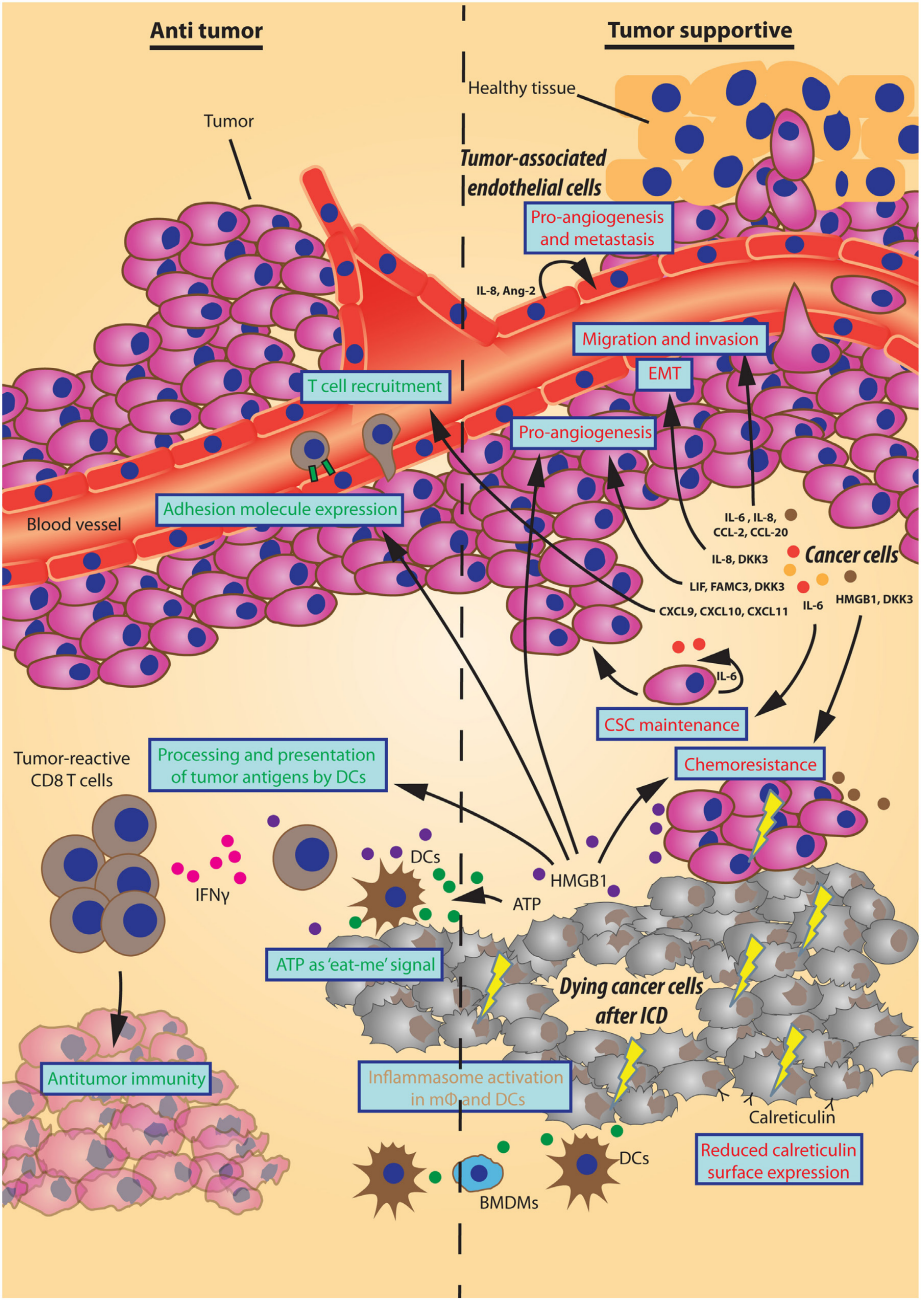
**Effects of autophagy-dependent secretion on tumor progression**. Schematic representation of autophagy-mediated secretory events that either inhibit or support tumor progression displayed here on the left (“Antitumor”) and right side (“Tumor supportive”), respectively. The different sources of autophagy-dependent secretory factors (*bold italic*) establish multiple effects on the tumor microenvironment (in blue boxes) by the designated factors. As such, factors that promote angiogenesis, invasion, a migratory phenotype, cancer stem cell (CSC) maintenance, or chemoresistance support tumor progression. Also, a reduced surface expression of calreticulin by cancer cells undergoing immunogenic cell death (ICD) hinders an immunogenic response. Oppositely, some factors have counteractive effects on tumor progression by improving immune cell adhesion or recruitment. Moreover, the “eat-me” signal ATP together with HMGB1/TLR4-mediated improved processing and presentation of tumor antigens by dendritic cells (DCs) promote interferon gamma (IFNγ)-producing T cells to aid antitumor immunity. The ATP/P2RX7-mediated activation of the inflammasome in macrophages (mφ) and DCs can have an array of effects of which the final inhibition/support of tumor progression may be context-dependent.

Autophagy has been considered as an important tumor suppressive process for cellular homeostasis by effectuating lysosomal degradation of the cells’ toxic constituents. Importantly, autophagy mediates an additional cellular feature, the trafficking, and release of specific proteins. These effects are important during physiological conditions (e.g., maintaining a barrier for infection by mucus and lysozyme secretion and waste secretion), but also mediate important effects in tumor progression (Figure 1). The autophagy-mediated secreted factors may stimulate cellular proliferation *via* auto- and paracrine signaling and establish a communicative tool between cells that can either stimulate or limit tumor progression. The autophagy-mediated release of DAMPs seems to be ICD inducer-dependent and polarize the TME toward a less immunesuppressive phenotype. Alternatively, tumors are characterized by promoting an immunosuppressed TME by cytokine signaling. Furthermore, autophagy-mediated secretory signaling promotes an invasive phenotype. An important note is that autophagy may convey direct or indirect effects on secretory events through formation of the mobilized vesicle, facilitation of anterograde trafficking or alterations in homeostasis, and/or autonomous cell signaling.

In conclusion, autophagy (or autophagy-related proteins) is an important cellular process that is more elaborate than solemnly a degradative pathway. It facilitates multiple secretory events that can promote tumor progression by limiting immunosurveillance and stimulating invasiveness and angiogenesis.

## Author Contributions

TK, MS, and KR reviewed literature, wrote the paper, drafted the outline, and approved content.

## Conflict of Interest Statement

The authors declare that the research was conducted in the absence of any commercial or financial relationships that could be construed as a potential conflict of interest.
